# Practical Considerations for Online Open Book Examinations in Remote Settings

**DOI:** 10.15694/mep.2020.000153.1

**Published:** 2020-07-31

**Authors:** Hui Meng Er, Vishna Devi Nadarajah, Pei Se Wong, Nilesh Kumar Mitra, Zabibah Ibrahim

**Affiliations:** 1 International Medical University

**Keywords:** Online examination, Open book examination, Remote, Health professional programmes

## Abstract

This article was migrated. The article was not marked as recommended.

The COVID-19 outbreak has led to lockdown of cities and restricted access to university campuses, and hence face-to-face delivery of education has been disrupted worldwide. In order to continue teaching, learning and assessment activities, academic institutions have embarked on online delivery and assessments using technology. Online open book examination is one of the tools considered during the crisis period to ensure that students’ progression in the academic programmes and graduation are not delayed. Its use is supported by literature evidences that show promotion of critical thinking and problem solving skills amongst students. The positive findings from our previous study on the impact of open book examinations on student performance and learning approach have encouraged us to implement online open book examinations in various health professional programmes in our institution during the COVID-19 pandemic. The objective of this paper is to share the practical tips for implementing online open book examinations remotely, in order to ensure the validity, reliability and fairness of the examinations.

## Introduction

The COVID-19 pandemic has resulted in lockdown of cities globally as public health measures to mitigate the spread of the coronavirus. Technology has rapidly been deployed by institutions to enable continuation of education delivery. This has accelerated the buy-in of online learning among the educators and students. Meanwhile, much attention has also been focussed on online assessments, particularly the platforms, tools and formats that can serve the assessment purpose and address its utility, i.e. validity, reliability, educational impact, acceptability and cost ( Van der Vleuten, 1996).

While closed book examinations promote students’ test preparation and deep learning ( Block, 2012; Durning, Dong, Ratcliffe, Schuwirth *et al*., 2016), open book examinations enhance their critical thinking and creative problem solving skills in an environment that simulates the real working scenario ( Johanns, Dinkens and Moore, 2017). In addition, open book assessments help students to develop competency in knowledge management ( Rowlands and Forsythe, 2006). Despite the availability of various online proctoring tools in the market, many have cautioned against their use in view of data privacy and confidentiality ( Thompson, 2020). Besides, some students may not have access to webcam that is required for online proctoring. These limit the conduct of closed book examinations remotely. Therefore, online open book examinations have been adopted by many academic institutions during this unprecedented period when campus access is restricted. The objective of this paper is to discuss the practical considerations for implementation of online open book examinations, based on our experience in conducting these examinations in various health professional programmes at our institution.

## Institutional Context and Programme Needs Analysis

**Figure 1.**
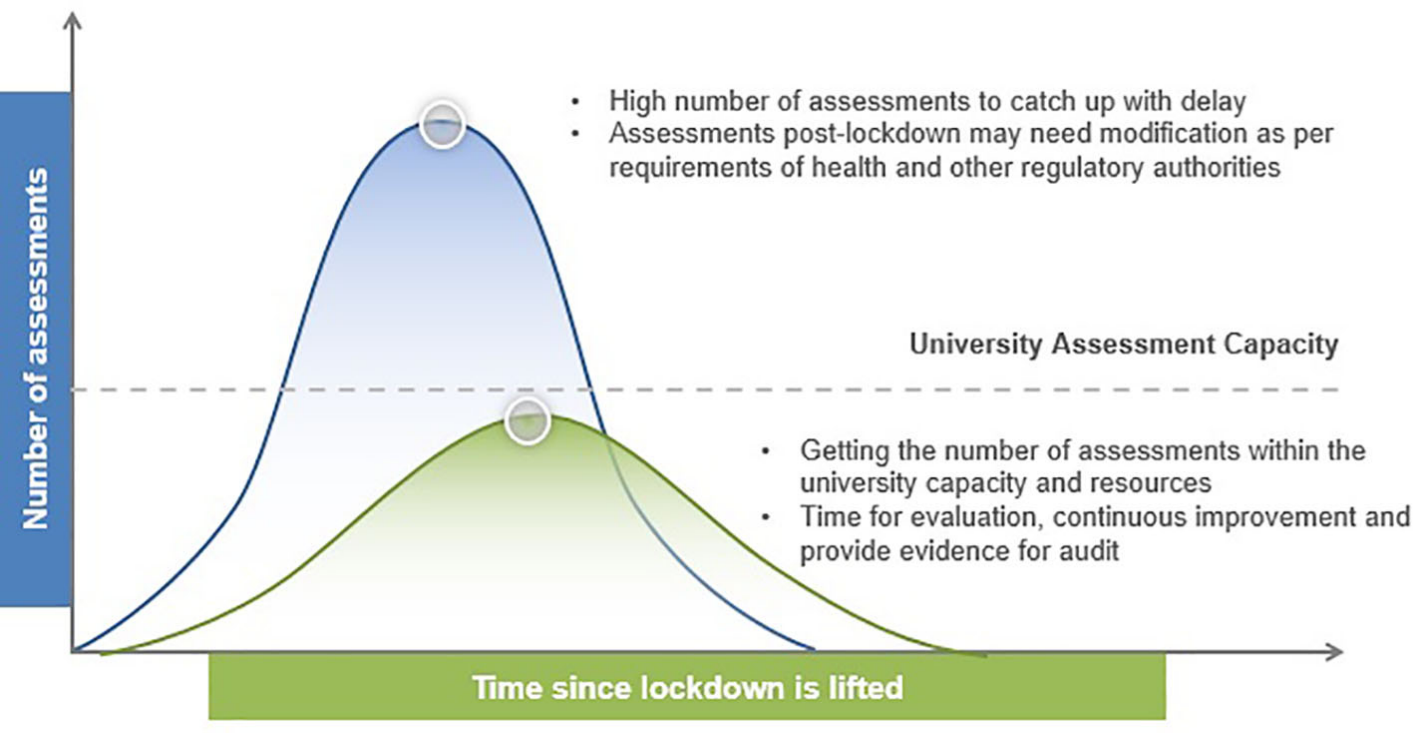
Managing the impact of examination postponement and post-lockdown modifications to campus activities

The decision to convert conventional examinations to online open book examinations should be made based on the institutional context and educational programme needs. These include the availability of a secure online assessment platform, administrative and logistic capacity of the institution, academic calendar, student assessment load as well as post-lockdown modifications to campus activities. As the COVID-19 situation continues to evolve, there is much uncertainty over the lockdown duration. Moreover, it is anticipated that conventional teaching, learning and assessment activities need to be modified post-lockdown in order to ensure physical distancing in the campus. Postponing the examinations to after lockdown is lifted will inevitably lead to increased workload of the examination administrators and logistic burden in scheduling assessment activities that may exceed the university capacity ( Figure 1). Examinations may need to be conducted in smaller groups and hence higher number of examination venues, extended examination schedules and quarantine procedures are necessary. It will also likely to delay students’ graduation and entry into the healthcare workforce. Assessment overload on the students is another consequence that could contribute to stress among the students. These implications have to be deliberated realistically against the challenges faced in converting to online open book examinations within a short period of time.

Figure 1 is based on the “Coronavirus Flattening the Curve PowerPoint Template” from SlideModel.com. The blue curve represents the consequence of postponement of examinations, while the green curve represents the effect of intervention through conduct of online open book examinations during and post-lockdown.

## Faculty Readiness

In open book examinations, students are allowed to refer to textbooks, online resources, or reference materials during the examinations ( Ramamurthy, Er, Nadarajah and Pook, 2016). Faculty training is necessary to ensure that the examinations are designed to test higher order thinking skills, e.g. applying, analysing, evaluating, and creating with resources ( Brightwell, Daniel and Stewart, 2004). Other skills can also be tested, such as locating information, using computer applications and conducting internet research using real world scenarios ( Johanns, Dinkens and Moore, 2017). These skills are indeed essential for healthcare professionals. However, some faculty may face challenges in preparing these types of questions within a short time, particularly in situations where clinical scenario or application based teaching is limited. To support this transition, university guidelines, webinars and personalised online faculty mentoring clinics for setting online open book examinations are highly recommended. On the other hand, it is important to recognise the limitations in assessing clinical and procedural skills using online open book examinations. Creativity makes it possible to assess some of these competencies, for example, by getting the students to demonstrate the skills via live streaming in anticipation that some modifications may be required.

## Students’ Readiness

Students have been reported to spend less time to prepare for open book examinations compared to closed book examinations due to misconception that the answers could be found in books ( Durning, Don, Ratcliffe, Schuwirth *et al*., 2016). As a consequence of inadequate preparation, some spent more time looking for answers rather than producing a quality answer ( Block, 2012). These findings have highlighted the importance of communicating to the students what online open book examinations are about and familiarising them with higher order thinking questions during teaching and formative assessments. Besides, the students should be informed in advance of the rules and regulations for online open book examinations including the consequences of academic misconduct.

Online open book examination relies on students’ computer hardware, software and internet connection during the examination. Evidences have shown that students could experience a greater cognitive load during online examinations compared to traditional paper-based examinations, as a result of having to deal with navigating technology in addition to answering the examination questions ( Cramp, 2019). This could lead to stress and anxiety. Hence, practice examinations are crucial for students to test their login to the online assessment platform, familiarise with the features of the platform and have practice opportunities with questions that require them to draw diagrams or plot graphs, either using computer software or by hand, followed by uploading to the answers for submission. Technical support should be available before and during the examinations. For example, if a student is disconnected from the online assessment platform during the examination due to internet connection problems, the technical support personnel can check the examination time remaining and the logs. Based on these reports, the examination coordinator can advise on the extra time allocation if needed. In situations where students face intermittent internet access, the asynchronous online mode should be considered, for example, by allowing the students to download the examination questions, work on the answers offline (without having to depend on internet connection) and submit the answers within the specified duration.

## Quality Assurance

It is important that the decision to convert conventional examinations to online open book examinations is made in compliance with the institutional governance in order to uphold the credibility and integrity of the academic programmes. With programme and course learning outcomes being the cornerstone, the design of online open book examinations should be guided by the assessment blueprint. Vetting of examination questions are particularly critical to ensure the validity, reliability, fairness and integrity of the examinations. Besides verifying the content, construct and concurrent validities, standard setting and psychometric analysis should continue to be practised for quality assurance. In addition, policies and standard operating procedures are required for the entire spectrum of work processes from preparing examination questions, vetting, uploading questions to the online assessment platform, authenticating test candidates, test taking, marking, to results processing, reporting and publishing. Not only will these ensure the examination efficiency, they safeguard the examination security which is a key concern since most of these activities are conducted online remotely. Besides the professional support staff of the examination office, technical staff from the Information Technology (IT) and E-Learning departments will also be involved in setting up the online examinations and providing technical assistance remotely during the examinations. Hence, the roles and responsibilities among the stakeholders should be clearly defined. Timely feedback from the stakeholders is crucial for continuous improvement.

## Adaptability and Agility

In preparation for the COVID-19 crisis, universities would have developed various business continuity plans (BCPs) to minimise impact to operations and ensure that teaching and learning activities remain viable, taking into consideration of personal and community safety. BCPs in this context will normally include plans to transition to online delivery and assessments, which are justified by transition risk assessment, needs prioritisation, resource readiness, communication strategies while maintaining academic standards and governance. Most of these BCPs are prepared at the institutional level, but they need to be contextualised to individual programme requirements to enable autonomy according to the varying context and communities they operate in. On the other hand, decisions related to public health and safety are usually made at the national level, resulting in varying external factors that are beyond the institution’s control. Nevertheless, the roll out of BCPs can be challenging in a constantly evolving situation like the COVID-19 pandemic. Frequent changes of plans are unavoidable and these can affect all stakeholders including the faculty, professional support staff and students. Reflecting on our experience, it can be concluded that while BCPs are useful guides, adaptability and agility among the stakeholders are crucial in a crisis situation. Factors that help to reduce the stress level of the stakeholders include data accessibility to guide decision making, preparedness of faculty for online delivery and assessments, availability of faculty development and mentoring, readiness and upskilling of professional support staff, as well as open and transparent communication and collaboration among all staff in a collegial manner with the common goal of ensuring positive student experiences during the challenging time.

## Conclusion

Online open book examination is a viable and relevant option for health professions education at times of uncertainty. The COVID-19 pandemic has increased the utility of this tool among the higher education institutions. However, it is important to have a systematic and collaborative approach for its implementation based on good practices, standards and evidences. Continuous studies on its validity, reliability and impact on learners should be carried out. The findings will help health professional educators to improve its implementation in order to ensure that the graduates are competent and work ready in an increasingly challenging healthcare environment.

## Take Home Messages

• The decision to convert conventional examinations to online open book examinations should be made based on the institutional context and educational programme needs.
• Faculty training and support for setting online open book examinations are highly recommended.
• Students should be familiarised with the conduct of online open book examinations through practice examinations.
• Quality assurance is important to ensure the validity, reliability and fairness of online open book examinations.
• Adaptability and agility among the stakeholders of online open book examinations are crucial in a crisis situation.

## Notes On Contributors

Professor Hui Meng Er is the Acting Dean of Teaching and Learning at the International Medical University, Malaysia.

ORCID iD: https://orcid.org/0000-0001-9835-4840.

Professor Vishna Devi Nadarajah is the Pro Vice Chancellor, Education and Institutional Development, at the International Medical University, Malaysia.

ORCID iD: https://orcid.org/0000-0002-7126-7189.

Dr Pei Se Wong is the Associate Dean of Teaching and Learning at the International Medical University, Malaysia.

ORCID iD: https://orcid.org/0000-0002-3958-0001.

Nilesh Kumar Mitra is the Acting Director of Learning Resources at the International Medical University, Malaysia.

ORCID iD: https://orcid.org/0000-0002-8487-4607.

Ms Zabibah Ibrahim is the Assistant Manager of E-Learning at the International Medical University, Malaysia.

ORCID iD: https://orcid.org/0000-0003-0555-2106.
