# Supplementary material for: Practical Considerations for Online Open Book Examinations in Remote Settings
Source: MedEdPublish (2016). 2021 Mar 19;9:153. Originally published 2020 Jul 31. [Version 2] doi: 10.15694/mep.2020.000153.2 (PMC10702684; doi:10.15694/mep.2020.000153.2)
Supplement: Peer review history

# Reviewer report: Reviewer response for version 2

**DOI:** 10.21956/mep.20272.r31468

**Published:** 2021-04-19

**Authors:** Gominda Ponnamperuma

**Affiliations:** 1 Faculty of Medicine, University of Colombo

This review has been migrated. The reviewer awarded 4 stars out of 5 This article provides useful practical information for any institute that plans to implement online learning. Out of all the sections, I particularly like the sections on quality assurance and student readiness. The open book assessment guides for faculty would also be quite useful for anyone planning to conduct open-book assessment. If the authors plan to revise this article further, I could offer the following suggestions.1. Since the objectives in the abstract and the main article slightly differ, it would be good to repeat the objective stated in the abstract (in exactly the same way) in the main article. This would ensure that both objectives contain 'validity, reliability and fairness'.2. The authors could develop a summary table that meshes the tips in relation to each of the sections (e.g. faculty readiness, student readiness, quality assurance, etc.) in rows against the assessment properties (e.g. validity, reliability, fairness) in columns.

Reviewer Expertise:

NA

No decision status is available

# Reviewer report: Reviewer response for version 2

**DOI:** 10.21956/mep.20272.r31469

**Published:** 2021-03-19

**Authors:** Ken Masters

**Affiliations:** 1 Sultan Qaboos University

This review has been migrated. The reviewer awarded 3 stars out of 5 I am pleased to see that the authors have made some attempt at addressing the issues raised in response to the first version, but the changes are not great. Although a Limitations section has been added, many of the other changes appear to be minor. For example, in the Abstract, apart from the addition of a single sentence (“While some successful progress…”), there appears to have been no change made, and it remains a lead-in to the paper rather than a summary of the paper.Almost the entire rest of the paper is identical with only minor alterations and insertions. So, while the paper’s value has been somewhat increased, I'm afraid it is rather a missed opportunity.

Reviewer Expertise:

NA

No decision status is available

# Reviewer report: Reviewer response for version 1

**DOI:** 10.21956/mep.19136.r27649

**Published:** 2020-11-03

**Authors:** Ken Masters

**Affiliations:** 1 Sultan Qaboos University

This review has been migrated. The reviewer awarded 2 stars out of 5 The paper aims at the practical considerations for online open book examinations in remote settings. While the paper does raise important issues, it is frustratingly short on practicalities. More details are given below, but, essentially, each section highlights the stuff that needs to be done, but gives very little practical indication on HOW it is to be done.• The Abstract gives a useful background to the paper, but should really be used to summarise the paper, so it would have been better if it had given a summary of the main points main in the paper.• In the Faculty Readiness, there is good information about the faculty being given training on open-book exams. Most readers who would benefit from this paper would be novices, and would probably not have access to such training, so it would be useful if the authors could supply some (preferably open-access) resources that get faculty up to speed as quickly as possible.• Similarly for students: are there more detailed resources that can be given to students to assist them with open-book exams?• Again (and I find myself repeating myself), the quality needs to be assured, and we need to set standards, yes, but how? Are there rubrics, recommendations, standard tools, etc.? How are these implemented?Perhaps the authors could produce a Version 2 of this paper in which they go into some more details about the practicalities and resources that can be used to achieve what has been outlined.

Reviewer Expertise:

NA

No decision status is available

# Reviewer report: Reviewer response for version 1

**DOI:** 10.21956/mep.19136.r27646

**Published:** 2020-10-28

**Authors:** Gominda Ponnamperuma

**Affiliations:** 1 Faculty of Medicine, University of Colombo

This review has been migrated. The reviewer awarded 4 stars out of 5 In these uncertain times, with on-site testing becoming almost impossible for most institutions and with the controversies that surround proctored online closed-book exams, open-book exams offer a breath of fresh air to the testing community. In this light, this article is a very useful eye-opener to the world of open-book testing. Authors should be congratulated for penning the important considerations when moving towards open-book testing. An important consideration that the authors could probably explore a bit more is 'standard setting'. Since the level of testing (i.e. the difficulty of items) in open-book exams is different, when compared with the closed-book exams, the standard setters may need to recalibrate their expectations of a passing (or a borderline) candidate.

Reviewer Expertise:

NA

No decision status is available

# Reviewer report: Reviewer response for version 1

**DOI:** 10.21956/mep.19136.r27643

**Published:** 2020-08-28

**Authors:** Richard Fuller

This review has been migrated. The reviewer awarded 4 stars out of 5 Open book testing or 'takeaway exams', which test skills right at the top of learning taxonomies has been an established component of assessment in other disciplines for some time. Open book questions are deliberately designed to test more complex questions that cannot be easily solved through recall, and employ wider learning strategies to access, synthesise and apply material from many sources. These questions require specific design and are very different from SBAs and SAQs - and may be more suitable for learners at later stages of programmes.They are a newer phenomenon in health professions education, although much has been written about them recently! Unfortunately, there has been a tendency to conflate them with online exams, and link them with concerns about cheating and probity. This has led to confusion by staff and students alike about online exams with closed book questions against the clock, vs truly open book takeaway type testing!A good introductory resource is from Twente University.https://www.utwente.nl/en/telt/online-lectures/remote-assessment/online-examination/open-book-exam/ This is a really useful review by colleagues from IMU and I commend the very practical focus on institutional readiness and most importantly, preparing students for a very different test format. Arguably, developing these formats further will help assessment be more 'authentic' and reflect the way that health professionals deal with complex patient issues and multiple sources of data (lab results, imaging, multi professional notes) - and how we work, and solve problems, as teams

Reviewer Expertise:

NA

No decision status is available

# Reviewer report: Reviewer response for version 1

**DOI:** 10.21956/mep.19136.r27647

**Published:** 2020-08-24

**Authors:** Viktor Riklefs

**Affiliations:** 1 Karaganda Medical University

This review has been migrated. The reviewer awarded 5 stars out of 5 Very useful article containing practical tips on what to consider when switching to online open-book quiz, probably the best option for remote settings. The authors give very practical advice on how to take into account the readiness of both students and teaching staff when designing the assessment, and how not to loose the quality. Many advices are given based on the own experience of the authors, which certainly adds value to the article.

Reviewer Expertise:

NA

No decision status is available

# Reviewer report: Reviewer response for version 1

**DOI:** 10.21956/mep.19136.r27645

**Published:** 2020-08-13

**Authors:** Ben Canny

**Affiliations:** 1 Monash University

This review has been migrated. The reviewer awarded 4 stars out of 5 Given that way that medical practitioners now practice (with a computer (smartphone) in our hands), I am consistently surprised that we have generally failed to embrace online, open internet examinations. Like many things during the pandemic, we have changed with great rapidity, and will wonder why the change took us so long. This article gives a great summary of how online open book examinations can be conducted, and may provide guidance of how to proceed once the "new normal" arrives.

Reviewer Expertise:

NA

No decision status is available

# Reviewer report: Reviewer response for version 1

**DOI:** 10.21956/mep.19136.r27642

**Published:** 2020-08-03

**Authors:** Hosam Eldeen Gasmalla

**Affiliations:** 1 Sudan International University

This review has been migrated. The reviewer awarded 4 stars out of 5 This article is one of many that in many ways puts an emphasis on programmatic assessment, it also shows that we need to adobt more diverse methods and tools.This tool "open-book examination" can also help focusing more on low-stakes formative assessments.

Reviewer Expertise:

NA

No decision status is available

# Reviewer report: Reviewer response for version 1

**DOI:** 10.21956/mep.19136.r27648

**Published:** 2020-07-31

**Authors:** Felix Silwimba

**Affiliations:** 1 University of Lusaka

This review has been migrated. The reviewer awarded 5 stars out of 5 this is a welcome alternative to student assessment. it is worth exploring.

Reviewer Expertise:

NA

No decision status is available

# Reviewer report: Reviewer response for version 1

**DOI:** 10.21956/mep.19136.r27644

**Published:** 2020-07-31

**Authors:** Hebat Allah A. Amin

**Affiliations:** 1 Department of Pathology, Helwan University, Faculty of Medicine

This review has been migrated. The reviewer awarded 5 stars out of 5 This is an interesting article addressing a real problem.I think sharing the experience through a case study or a Step by Step Guide would be more fruitful and allow the dissimination of the experience. Thanks for the important topic.

Reviewer Expertise:

NA

No decision status is available
